# Hallmarks of epithelial to mesenchymal transition are detectable in Crohn’s disease associated intestinal fibrosis

**DOI:** 10.1186/s40169-015-0046-5

**Published:** 2015-02-07

**Authors:** Michael Scharl, Nicole Huber, Silvia Lang, Alois Fürst, Ekkehard Jehle, Gerhard Rogler

**Affiliations:** Division of Gastroenterology and Hepatology, University Hospital Zürich, Rämistrasse 100, 8091 Zurich, Switzerland; Zurich Center for Integrative Human Physiology, University of Zürich, Zürich, Switzerland; Department of Surgery, Caritas-Hospital St. Josef, Regensburg, Germany; Department of Surgery, Oberschwaben-Klinik, Ravensburg, Germany

**Keywords:** Crohn’s disease, Epithelial-mesenchymal transition, Intestinal fibrosis

## Abstract

**Background:**

Intestinal fibrosis and subsequent stricture formation represent frequent complications of Crohn’s disease (CD). In many organs, fibrosis develops as a result of epithelial to mesenchymal transition (EMT). Recent studies suggested that EMT could be involved in intestinal fibrosis as a result of chronic inflammation. Here, we investigated whether EMT might be involved in stricture formation in CD patients.

**Methods:**

Human colonic tissue specimens from fibrotic areas of 18 CD and 10 non-IBD control patients were studied. Immunohistochemical staining of CD68 (marker for monocytes/macrophages), transforming growth factor-β_1_ (TGFβ_1_), β-catenin, SLUG, E-.cadherin, α-smooth muscle actin and fibroblast activation protein (FAP) were performed using standard techniques.

**Results:**

In fibrotic areas in the intestine of CD patients, a large number of CD68-positive mononuclear cells was detectable suggesting an inflammatory character of the fibrosis. We found stronger expression of TGFβ_1_, the most powerful driving force for EMT, in and around the fibrotic lesions of CD patients than in non-IBD control patients. In CD patients membrane staining of β-catenin was generally weaker than in control patients and more cells featured nuclear staining indicating transcriptionally active β-catenin, in fibrotic areas. In these regions we also detected nuclear localisation of the transcription factor, SLUG, which has also been implicated in EMT pathogenesis. Adjacent to the fibrotic tissue regions, we observed high levels of FAP, a marker of reactive fibroblasts.

**Conclusions:**

We demonstrate the presence of EMT-associated molecules in fibrotic lesions of CD patients. These findings support the hypothesis that EMT might play a role for the development of CD-associated intestinal fibrosis.

**Electronic supplementary material:**

The online version of this article (doi:10.1186/s40169-015-0046-5) contains supplementary material, which is available to authorized users.

## Background

Though most of IBD patients initially present with an inflammatory pathology, due to the longstanding and chronically relapsing disease, severe complications, such as stenosis, strictures or fistulae occur [1]. With respect to Crohn’s disease (CD), about 70% of patients suffer from fistula and stenosis during their disease course and at least 60% require surgery at least once within 20 years following their initial diagnosis [2]. About one third of CD patients require surgery due to intestinal fibrosis or the resulting complications, such as intestinal obstruction, during their disease course. However, this often also does not provide a definite solution and inflammation and restenosis frequently re-occur [3]. Of note, there are distinct differences between intestinal fibrosis in UC and CD patients: While in UC, the colonic mucosa and sub-mucosa is affected, in CD the fibrosis is transmural, mainly of the small and/or large bowel, including submucosa, muscle layer and serosa, but excluding the mucosa [4]. In contrast to CD, surgery due to fibrotic complications is a rare event in UC patients, whereas fibrotic events occur at least evenly. A key problem with respect to inflammation-associated intestinal fibrosis is the fact that anti-inflammatory strategies, such as anti-TNF antibodies or immunosuppressive medications, are not effective in preventing fibrosis and that no specific anti-fibrotic medical therapy exists [4].

Current hypothesis suggests that the first step of intestinal fibrosis is a tissue damage caused by a chronic inflammatory state. Subsequently, activated fibroblasts are recruited to the sites of inflammation to induce wound healing and, finally, fibrosis results due to excessive deposition of extracellular matrix (ECM) [5,6]. Recently, is has been demonstrated that miR-200b is involved in intestinal fibrosis in CD patients [7]. Using an animal model of chronic intestinal inflammation it was shown that CD-associated intestinal fibrosis might be the result of epithelial-to-mesenchymal transition (EMT) [8]. EMT is a common process that plays a pivotal role for embryogenesis, organ development, tissue regeneration and wound healing [9,10]. However, EMT is also involved in organ fibrosis as well as cancer progression and metastasis [9,11]. During EMT, epithelial cells lose their polarized phenotype and convert into mesenchymal-like myofibroblasts which is indicated by co-expression of epithelial markers, such as E-cadherin or cytokeratines 8 and 20, and mesenchymal markers, such as α-smooth muscle actin (α-SMA) or vimentin, in EMT cells [10,12]. This way, EMT represents one of the main sources of activated fibroblasts in many organ systems and its most powerful mediator *in vitro* and *in vivo* is transforming growth factor beta (TGFβ) [11-15].

Here, we demonstrate the presence of many EMT-related proteins in fibrotic areas of intestinal tissue specimen derived from CD patients. Our data suggest that EMT is essentially involved in the pathogenesis of CD-associated intestinal fibrosis. This finding might open the avenue for new and more effective treatment options for intestinal fibrosis.

## Methods

### Patients

Colonic tissue specimens were obtained from fibrotic areas of 18 CD patients (mean age 44 ± 4 years) or from the intestinal mucosa of 10 non-IBD control patients (mean age 62 ± 5 years). Intestinal samples were derived from male and female patients (please see Additional file 1: Table S1 + 2 for further details). Tissue specimens were collected from endoscopic biopsies or surgical specimens from CD patients or from non-IBD patients who underwent endoscopy because of colon cancer screening, respectively. Tissue specimens were immediately transferred into 4% formalin and stored at 4°C until further analysis. Written informed consent was obtained before specimen collection and studies were approved by the Cantonal Ethics Committee of the CAnton Zürich, Switzerland

### Immunohistochemistry

We performed immunohistochemical studies on formalin-fixed, paraffin-embedded tissue specimens using a peroxidase based method with diaminobenzidine (DAB) chromogen as described previously [16]. Following incubation of tissue samples with xylol and descending concentrations of ethanol, antigens were retrieved using citrate buffer, pH 6.0 (DAKO, Glostrup, Denmark) for 30 min at 98°C. Endogenous peroxidases were deleted by incubation with 0.9% hydrogen peroxide for 15 min at room temperature (RT) and blocking was performed using 3% BSA for 1 h at RT. Antibodies were then applied in an optimal concentration overnight in a wet chamber. Rabbit anti-CD68 (Abcam, Cambridge, UK), rabbit anti-β-catenin (Cell Signalling), rabbit anti-TGFβ (Santa Cruz, Santa Cruz, CA), rabbit anti-SLUG (Abcam), rabbit anti-E-cadherin (Cell Signalling, Danvers, MA), rabbit anti-α-smooth muscle actin (SMA; Abcam) and rabbit anti-fibroblast activating protein (FAP; Abcam) antibodies were obtained from the sources noted. EnVision^+^ System-HRP-Labelled Polymer (DAKO) was used as a secondary antibody and was applied for 1 h at RT. Antibody binding was visualized using Liquid DAB^+^ Substrate Chromogen System (DAKO). Samples were counterstained with hematoxylin, incubated in ascending concentrations of ethanol and xylol solutions and finally mounted. For microscopic assessment, an AxioCam MRc5 (Zeiss, Jena, Germany) camera on a Zeiss Axiophot microscope (Zeiss) wa used. Image analysis was performed using AxioVision Release 4.7.2 software (Zeiss).

## Results

### Fibrotic lesions show CD68-positive mononuclear cells

**Figure 1:**
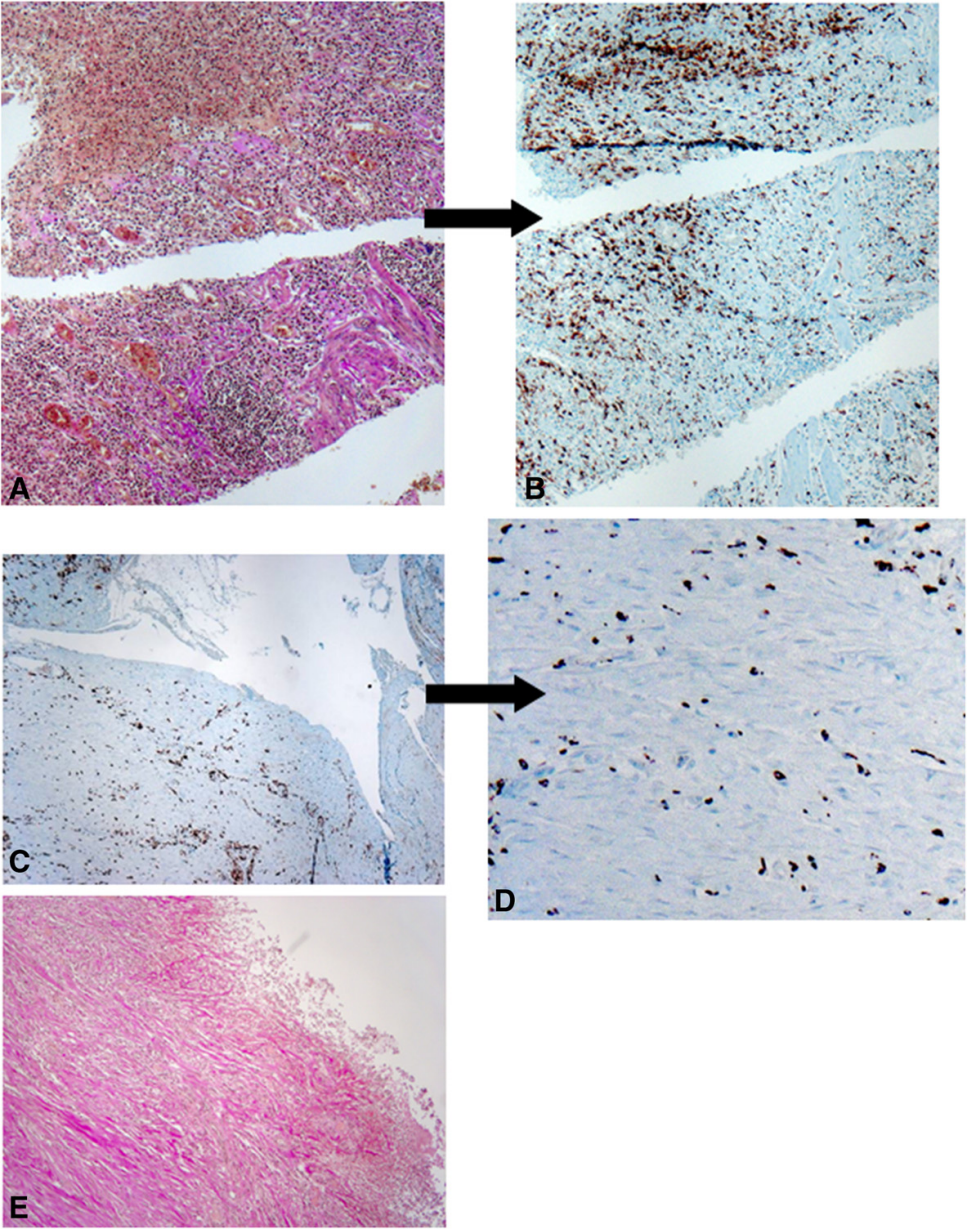
**CD68-positive mononuclear cells are present in fibrotic lesions. (A)** Colonic tissue specimens from CD patients with active disease feature a large amount of collagen fibers indicative for fibrosis as visualized by van-Gieson staining. **(B)** Same area as in (A) features strong expression of CD68, a marker of activated monocytes/macrophages. **(C)** Fibrotic regions in the intestine of CD patients feature strong expression of CD68. **(D)** Enlarged section from **(C)** featuring CD68-positive mononuclear cells. **(E)** Representative section from **(C)** visualizing collagen fibers by van-Gieson staining. Magnification: 10-fold or 40-fold, respectively. All analysed patient samples showed comparable staining characteristics when compared to samples from non-affected controls.

Sites of severe inflammation often feature increased numbers of activated fibroblasts. These cells, also called myofibroblasts, play an important role for wound healing what sometimes results in excessive production of ECM finally leading to fibrosis. To identify fibrotic areas we visualized collagen fibers using van Gieson staining, while we confirmed the presence of inflammatory cells in fibrotic areas by CD68 staining, which is a well-established marker for human monocytes and macrophages [17]. As expected, we found a large number of CD68 positive cells in and around fibrotic areas of colonic tissue samples from CD patients (Figure 1), what correlates with the hypothesis that intestinal fibrosis is associated with inflammation.

### TGFβ is strongly expressed in fibrotic colonic CD tissue

**Figure 2:**
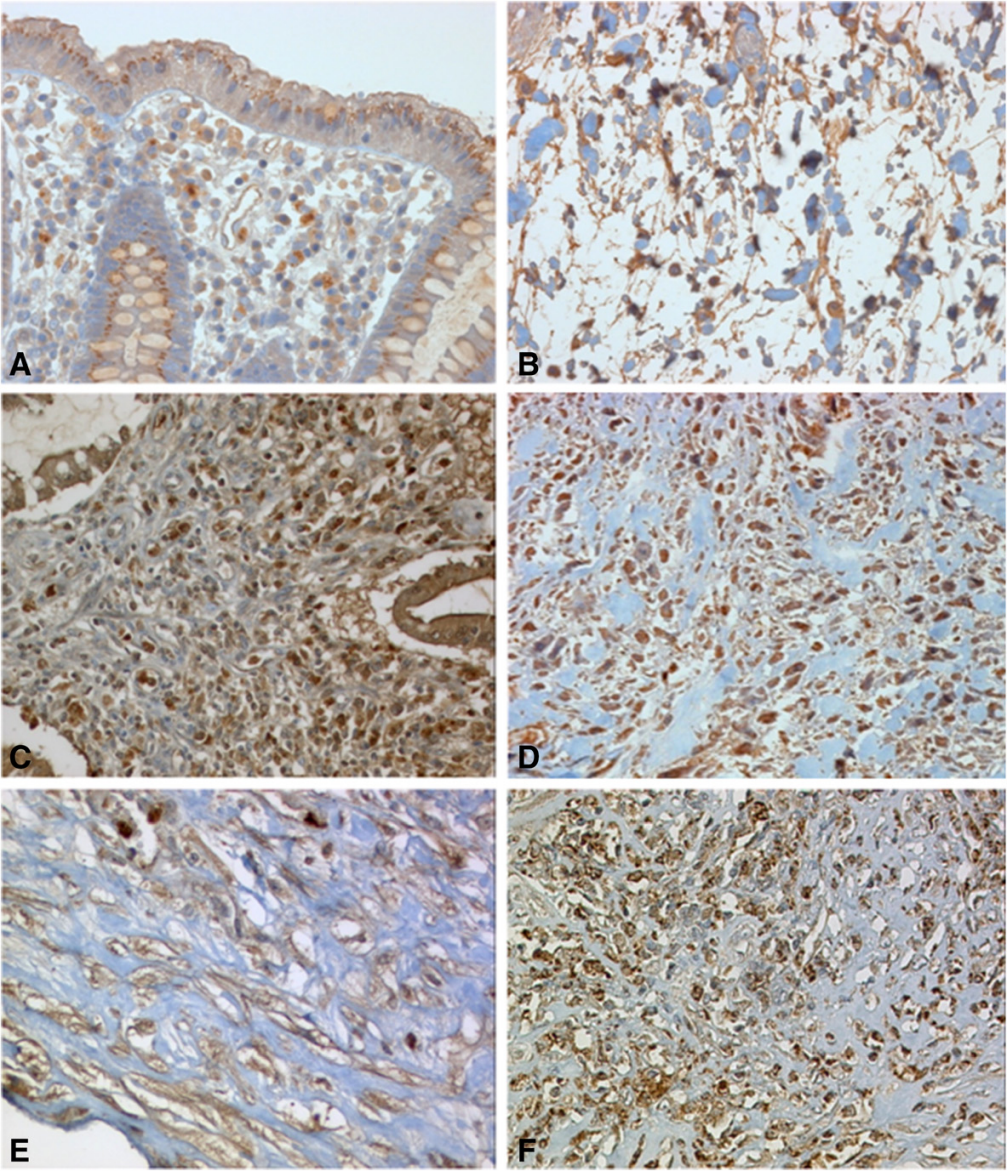
**Expression of TGFβ**_**1**_ **in fibrotic lesions. (A + B)** In representative samples from two different non-IBD control patients, the epithelial layer showed a considerable staining intensity for TGFβ_1_, while the staining in sub-mucosal layers was only slightly detectable. **(C)** In CD patients with intestinal fibrosis, expression of TGFβ_1_ in the epithelial layer was strongly detectable. **(D-F)** In fibrotic areas, sub-mucosal TGF-β1 staining is strongly detectable. Magnification: 20-fold or 40-fold, respectively. All analysed patient samples showed comparable staining characteristics when compared to samples from non-affected controls.

TGFβ is widely regarded as the most powerful inducer of EMT *in vitro* and *in vivo*. Since the most important TGFβ isoform in humans is represented by TGFβ_1_, we assessed expression of this particular molecule in our study. First we investigated TGFβ_1_ expression in non-IBD control patients without fibrosis. Here, the epithelial layer showed a considerable staining intensity for TGFβ_1_, while the staining in sub-mucosal layers was only slightly detectable (Figures 2A + B). In contrast, in CD patients with intestinal fibrosis, expression of TGFβ_1_ in the epithelial layer was stronger than in control patients (Figure 2C). Further TGFβ_1_ expression in submucosal layers and in particular in fibrotic areas was clearly more in CD patients than in control patients (Figures 2D-F). This difference could be observed in all of the tissue layers from the lamina propria to the serosal layer.

### Nuclear localisation of β-catenin in fibrotic areas

**Figure 3:**
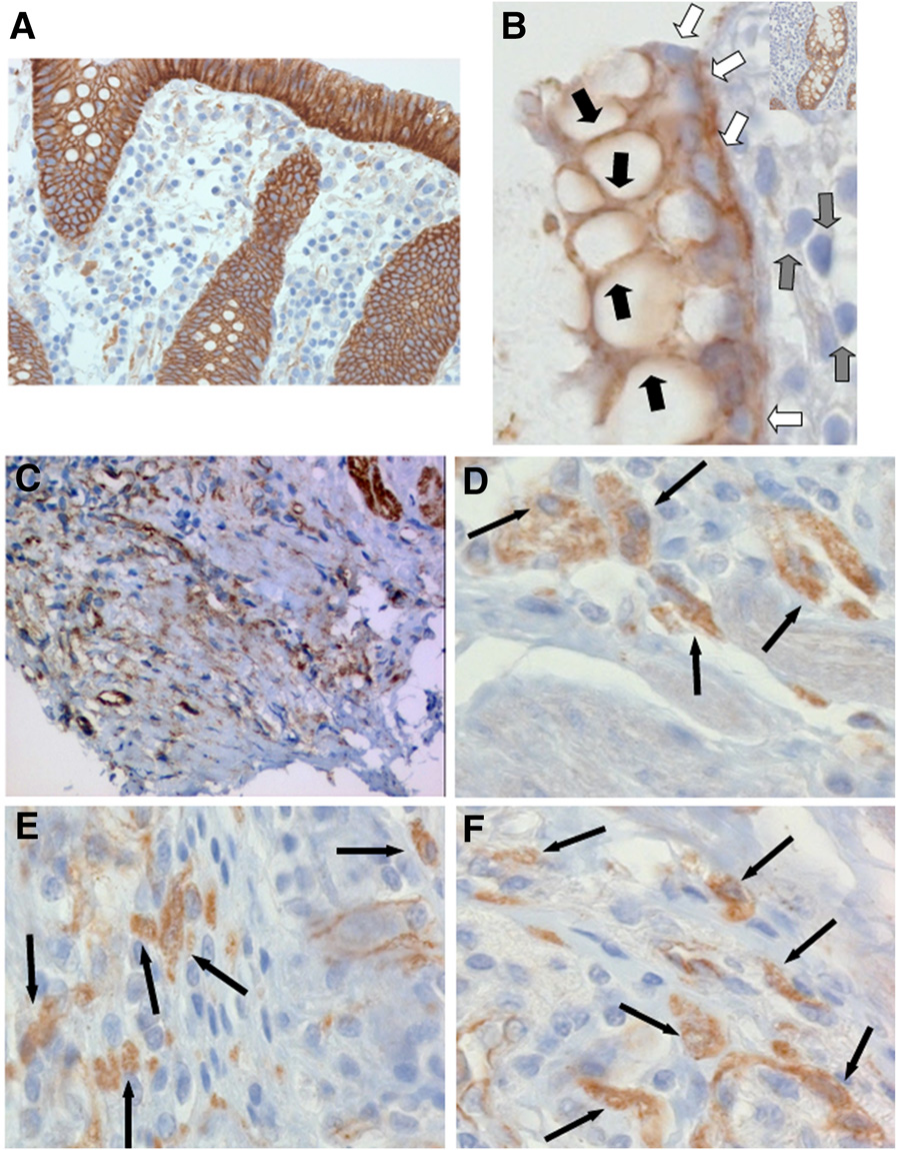
**Expression of β-catenin in fibrotic lesions. (A + B)** In tissue samples from non-IBD control patients, the intestinal epithelial cells featured a strong staining for β-catenin in the cell membrane (black arrows) while the nuclei did not show any significant β-catenin staining (white arrows). In sub-mucosal layers almost no β-catenin was detectable (grey arrows). **(C-F)** In stenotic areas of CD patients clearly more cells featured nuclear β-catenin staining (black arrows) indicating transcriptionally active, meaning nuclear, β-catenin. Magnification: 10-fold, 40-fold or 100-fold, respectively. All analysed patient samples showed comparable staining characteristics when compared to samples from non-affected controls.

A well-established marker for the onset of EMT is nuclear localisation of β-catenin. We first assessed β-catenin staining in the epithelial layer. In tissue samples from control patients, all of the intestinal epithelial cells featured a strong staining for β-catenin in the cell membrane while the nuclei did not show any significant β-catenin staining. In sub-mucosal layers almost no β-catenin was detectable at all (Figures 3A + B). In CD patients membrane staining of β-catenin was generally weaker than in control patients and more cells featured nuclear staining indicating transcriptionally active β-catenin (Figures 3C-F). In fibrotic areas, nuclear expression of β-catenin was clearly higher in fibroblasts than in control patients and fibrotic areas featured hereby an even stronger nuclear staining intensity for β-catenin (Figures 3C-F). The increased number of cells featuring nuclear translocation of β-catenin was also demonstrated by statistical analysis of nuclear β-catenin positive cells per a well-defined field-of-view in a representative subgroup of patients (Additional file 1: Figure S1). These observations, loss of cell membrane-bound β-catenin and increased nuclear localisation indicative for transcriptionally active β-catenin in fibroblasts, are characteristic observations for EMT and further support the observation that EMT might be involved in CD-associated fibrosis.

### SLUG transcription factor is upregulated in CD-associated fibrosis

**Figure 4:**
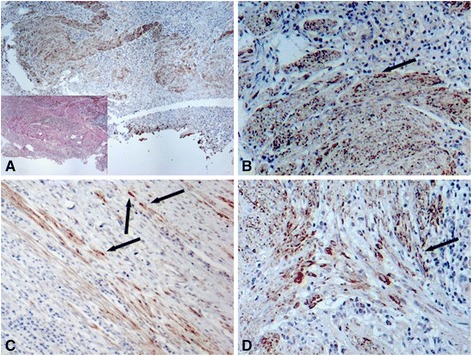
**The transcription factor SLUG is expressed in the nuclei of mesenchymal cells in fibrotic areas. (A-D)** Representative images demonstrate considerable SLUG staining in fibroblast-like cells of fibrotic areas of colonic tissue specimens derived from CD patients. SLUG is visible in the nuclei of fibroblast-like cells (black arrows) indicative for transcriptionally active SLUG protein. The insert in **(A)** demonstrates van-Gieson staining. Magnification: 10-fold or 40-fold, respectively. All analysed patient samples showed comparable staining characteristics when compared to samples from non-affected controls.

We next investigated the expression of Snail transcription factor family members, namely SLUG and Snail1 in fibrotic intestine of CD patients. We found strong SLUG staining in fibroblast-like cells of fibrotic areas of colonic tissue specimens derived from CD patients. Of notice, SLUG was often visible in the nuclei of fibroblast-like cells indicative for transcriptionally active SLUG protein (Figure 4A-D). Increased expression and nuclear translocation of SLUG are often correlated with the onset of EMT and our finding provides therefore a possible mechanistic explanation how EMT-associated events in fibrotic areas could be mediated on a transcriptional level. Slug staining was almost completely absent in non-IBD control patients (data not shown). Interestingly, the transcription factor SNAIL1, was neither detectable in non-IBD control patients nor in fibrotic regions of CD patients (data not shown).

### FAP is strongly expressed around areas of CD-associated fibrosis

**Figure 5:**
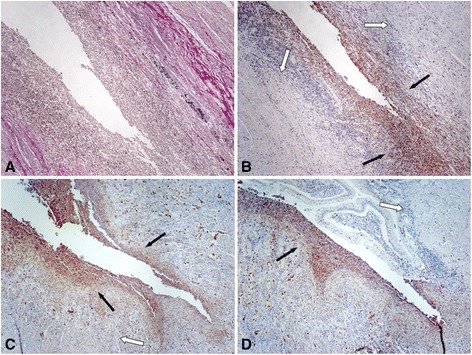
**FAP expression is strongly detectable in myofibroblasts adjacent to fibrotic areas.** Representative images show **(A)** van-Gieson staining and **(B)** corresponding FAP staining in fibrotic tissue samples derived from CD patients. **(B-D)** FAP staining is strongly detectable in myofibroblasts adjacent to the fibrotic areas (black arrows), while FAP staining in myofibroblasts within fibrotic areas is limited (white arrows). Magnification: 10-fold. All analysed patient samples showed comparable staining characteristics when compared to samples from non-affected controls.

A number of studies indicated a role for FAP in the pathogenesis of chronic inflammatory and fibrotic conditions, such as liver cirrhosis. Since CD-associated fibrosis also occurs in response to chronic inflammation of the intestinal wall, we investigated whether FAP, a marker of fibroblast activation, would be expressed in or around fibrotic areas in the intestine of CD patients. Figure 5 demonstrates that FAP was only weakly expressed in the fibrotic areas. However, we found a strong, FAP-positive staining in fibroblasts adjacent to the fibrotic areas.

### Expression of epithelial and mesenchymal markers in fibrotic intestinal CD tissue

**Figure 6:**
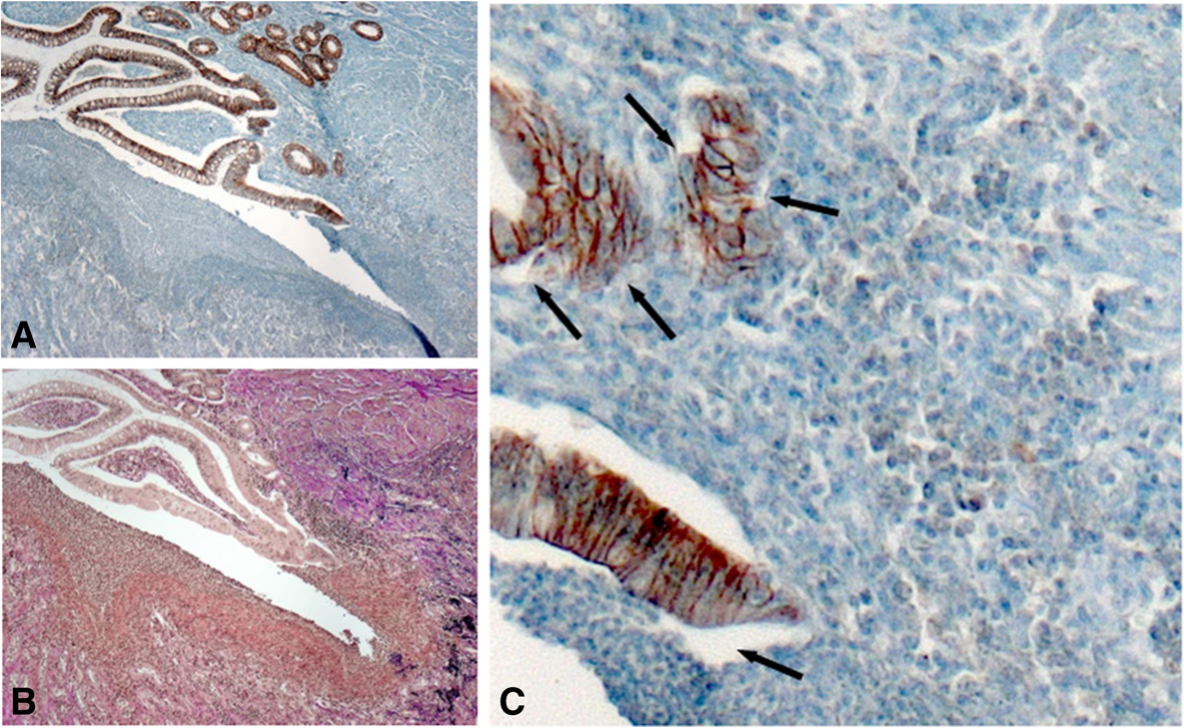
**Subepithelial cells in and around fibrotic areas express E-cadherin.** Representative images show E-cadherin **(A; C)** and van-Gieson **(B)** staining in corresponding tissue sections. Black arrows point onto E-cadherin positive cells. Magnification: 10-fold or 40-fold, respectively. All analysed patient samples showed comparable staining characteristics when compared to samples from non-affected controls.

**Figure 7:**
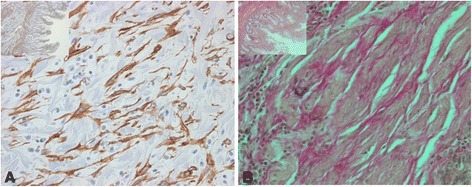
**Subepithelial cells in and around fibrotic areas express α-SMA.** Representative images show α-SMA **(A)** and van-Gieson **(B)** staining in corresponding tissue sections. Magnification: 10-fold or 40-fold, respectively. All analysed patient samples showed comparable staining characteristics when compared to samples from non-affected controls.

We next assessed the expression of E-cadherin which is usually located in adherens junctions of epithelial cell layers, connecting adjacent cells. We found strong E-cadherin staining in the intestinal epithelial layer (Figure 6A). However, we also detected expression of E-cadherin in a number of subepithelial cells in and around fibrotic areas (Figure 6B + C). Further, we found a large number of α-SMA positive cells in fibrotic areas of intestinal tissue specimens from CD patients (Figures 7A-B). This co-incidence of mesenchymal and epithelial markers in areas of fibrotic tissue strongly supports an involvement of EMT in the pathogenesis of CD-associated fibrosis.

## Discussion

CD-associated fibrosis and the resulting clinically relevant stenosis and strictures represent a common and severe complication in CD patients. To date, conventional options for prevention or treatment of such fibrotic complications are insufficient and even surgery is often only a temporarily beneficial approach. The limitation in available treatment options is mainly due to the fact that the pathogenesis of CD-related fibrosis is only poorly understood. Here, we demonstrate the presence of EMT-associated events in fibrotic areas of colonic tissue specimens derived from CD patients.

A characteristic feature of EMT is the translocation of β-catenin from the cytosol, where it connects E-cadherin to the actin cytoskeleton, into the nucleus to regulate gene expression. During EMT, β-catenin translocates from the cell membrane into the cytoplasm indicative for the disintegration of the epithelial zonulae adherentes. Further, the cytoplasmic pool of β-catenin translocates into the nucleus and initiates the expression of EMT-associated genes, such as α-SMA, vimentin or TGFβ [18,19]. TGFβ has been shown to regulate expression and activity of the Snail transcription factor family member, SLUG, via β-catenin in epithelial cell systems and SLUG has also been implicated in the pathogenesis of EMT via down-regulation of E-cadherin [20]. Characteristically, activated fibroblasts express FAP and presence of FAP has been implicated in the pathology of cancers, chronic inflammatory disorders, fibrosis and other pathologies indicating possible roles for FAP in facilitating cell invasion and growth [21].

Though EMT has already been associated with the pathogenesis of fibrosis in many systems, such as kidney, liver or lung [11], the evidence for EMT being involved in the development of intestinal fibrosis in CD patients is lacking. A recent study by Flier et al. identified EMT as a source of fibroblasts in the TNBS model of inflammation-associated intestinal fibrosis. They demonstrated that intra-rectal administration of TNBS to mice induces inflammation and fibrosis that is histological and immunologically similar to human CD. The fibrotic areas in these animals featured a large amount of cells expressing both, epithelial and mesenchymal markers, indicative for the onset of EMT. In particular, fibrotic areas revealed significantly more fibroblasts being α-SMA-positive as well as being positive for both, the epithelial cell marker E-cadherin and the fibroblast marker, fibroblast-specific protein 1 (FSP1) [8]. However, the observation period of these animals was certainly limited when compared to year-long duration of fibrogenesis in humans. Additionally, these mice did not develop strictures that display one of the most severe complications of human intestinal fibrosis. In intestinal tissue samples derived from three patients suffering from either active UC or CD, they showed co-localisation of α-SMA and E-cadherin in colonic crypts [8]. Nevertheless, this patient number was certainly too small for demonstrating finally conclusive results and the patients were also not featuring strictures as a complication of intestinal fibrosis.

In our new animal model of intestinal fibrosis occurring after heterotopic transplantation of small bowel resections in rats we found rapid loss of crypt structures which was followed by lymphocyte infiltration and obliteration of the intestinal lumen by fibrous tissue. Loss of intestinal epithelium was demonstrated by lack of cytokeratines while collagen expression was increased with time after transplantation. Interestingly, lumen obliteration was connected with increased expression of factors associated with EMT such as β_6_ integrin, IL-13 and TGFβ. The myofibroblast phenotype in fibrotic areas was demonstrated by presence of the mesenchymal markers α-SMA smooth muscle actin and vimentin in the obliterated lumen. These observations demonstrate that a variety of histologic and molecular features of fibrosis associated EMT can be observed in the heterotopic intestinal grafts [22]. Therefore, the data obtained in our new animal model so far fully confirmed our data obtained in human samples suggesting that the observations made in the animal model might actually reflect real fibrosis. This of particular interest, since it not only underlines the relevance of our data presented in this manuscript, but also gives us the possibility to perform further research using a reproducible animal model. This might finally critically contribute to a better understanding of the complex pathogenesis of intestinal fibrosis and the development and validation of new therapeutic options.

Thus, the observations presented here are in good accordance with other findings from our laboratory, demonstrating that EMT also is present in human CD and contributes to the pathogenesis of CD-associated fistulae [16,23-25]. Of note, intestinal fistulae are commonly surrounded by areas featuring fibrotic lesions. However, neither the findings in the animal models nor our previous data provided conclusive evidence that EMT might be involved in the pathogenesis of intestinal fibrosis in CD patients.

Here, we demonstrated that areas of intestinal fibrosis in CD patients feature EMT-associated gene expression events using colonic tissue specimens from fibrotic areas of CD patients. We found significant staining of TGFβ as well as of the EMT-associated transcription factor SLUG in fibrotic areas. Additionally, we detected nuclear localisation of β-catenin as a prominent feature of fibrotic regions. All of these molecules are associated with the onset of EMT. In particular, in intestinal epithelial cells TGFβ can induce SLUG expression and activation (meaning nuclear localisation) via β-catenin. Further, activated SLUG as well as activated (meaning nuclear) β-catenin play an important role for the downregulation and disassembling of E-cadherin [12,15,18-20]. All of these events (expression of TGFβ and nuclear localisation of SLUG and β-catenin) are characteristic for EMT and were visible in human tissue samples featuring a CD-associated fibrosis.

Of interest, FAP staining was strongly detectable in fibroblasts adjacent to the fibrotic areas. Though FAP expression is absent in most adult tissues, it is expressed under chronic inflammatory and fibrotic conditions as well as in certain epithelial tumours. On a functional level, FAP features dipeptidyl- and endopeptidase activity and seems to be involved in tumour cell proliferation [21,26]. A recent study even suggested that FAP inhibition might be beneficial for treating epithelial-derived tumours, since pharmacologic targeting of FAP inhibited tumour stromagenesis by affecting integrin-mediated signalling and led to a decreased recruitment and overall number of myofibroblasts [27]. Therefore, one might speculate, whether FAP could be a potential target for the treatment of CD-associated intestinal fibrosis.

## Conclusions

In summary, we have demonstrated the presence of EMT-associated events in fibrotic areas in the colon of CD patients. These findings suggest that EMT could play a certain role for the pathogenesis of CD-associated intestinal fibrosis. Therefore our study might help to provide the rationale background for further investigations unravelling the exact pathomechanisms of intestinal fibrosis what could finally help to develop more effective therapeutic options for the treatment of CD-associated intestinal fibrosis.

## Additional file

Additional file 1:
**Supplemental methods and figures.**

## Abbreviations

α-SMA: alpha smooth muscle actin
CD: Crohn’s disease
ECM: extra-cellular matrix
EMT: epithelial-to-mesenchymal transition
FAP: fibroblast activating protein
TGFβ: transforming growth factor beta
