# Supplementary material for: Hallmarks of epithelial to mesenchymal transition are detectable in Crohn’s disease associated intestinal fibrosis
Source: Clin Transl Med. 2015 Feb 7;4:1. doi: 10.1186/s40169-015-0046-5 (PMC4384762; doi:10.1186/s40169-015-0046-5)
Supplement: Additional file 1: — Supplemental methods and figures. [file 40169_2015_46_MOESM1_ESM.doc]

**SUPPLEMENTAL MATERIAL**

**1. Supplementary tables**

**Age Gender Diagnosis Origin of tissue specimens**

20 male Crohn’s disease Sigma

66 female Crohn’s disease Ileo-Ascendens-Anastomosis

79 male Crohn’s disease Terminal Ileum

31 male Crohn’s disease Ileum

47 male Crohn’s disease Ileo-Ascendens-Anastomosis

32 male Crohn’s disease Ileocaecal region

67 female Crohn’s disease Ileocaecal region

49 female Crohn’s disease Ileocaecal region

57 male Crohn’s disease Small intestine

51 male Crohn’s disease Terminal Ileum

31 male Crohn’s disease Ileum

34 female Crohn’s disease Colon transversum

31 male Crohn’s disease Terminal Ileum

50 male Crohn’s disease Ileum

51 male Crohn’s disease Ileum

25 male Crohn’s disease Terminal Ileum

34 male Crohn’s disease Ileum

33 male Crohn’s disease Ileum

**Supplementary table 1: Characteristics of Crohn’s disease patients**. Intestinal samples from 18 patients with CD were analyzed.

**Age Gender Diagnosis Origin of tissue specimen** 52 male Colorectal adenocarcinoma Rectum

81 male Adenoma of the colon Colon transversum

85 female Adenoma of the colon Colon descendens

34 female Endometriosis Ileocoecal region

66 male Colorectal adenocarcinoma Small intestine

54 female Adenoma of the colon Colon descendens

62 male Colorectal adenocarcinoma Colon transversum

73 male Adenoma of the colon Colon descendens

59 female Adenoma of the colon Ileocoecal region

51 female Colorectal adenocarcinoma Colon descendens

**Supplementary table 2: Characteristics of non-IBD control patients**. Intestinal samples from 10 non-IBD control patients were analyzed. Samples were derived from patients that underwent colon surgery because of tumours. Analyzed tissue specimen were obtained from tumour-free edge of the excised area.

**2. Supplementary data**

Representative images from 3 Crohn’s disease patients:


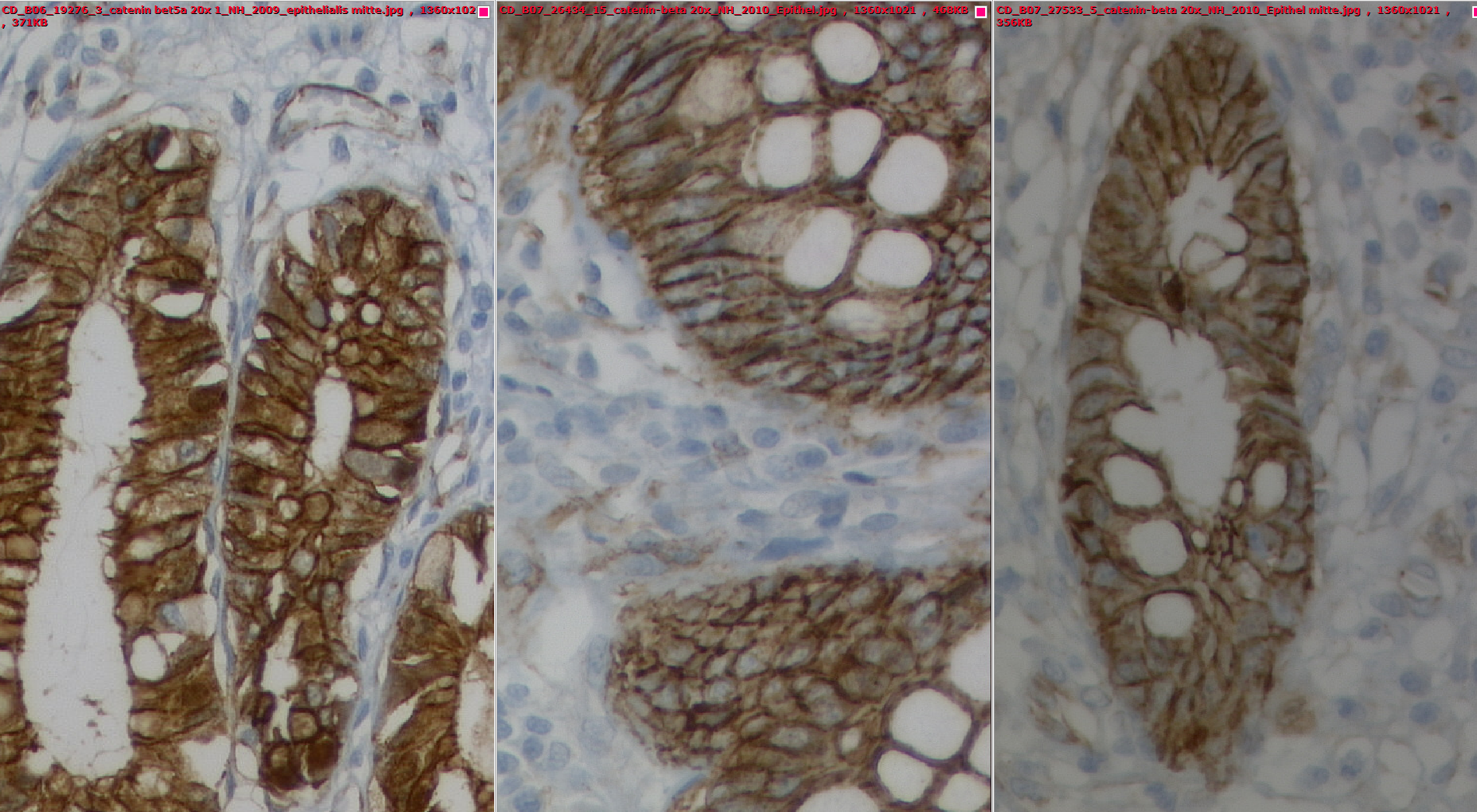


Representative images from 2 control patients:


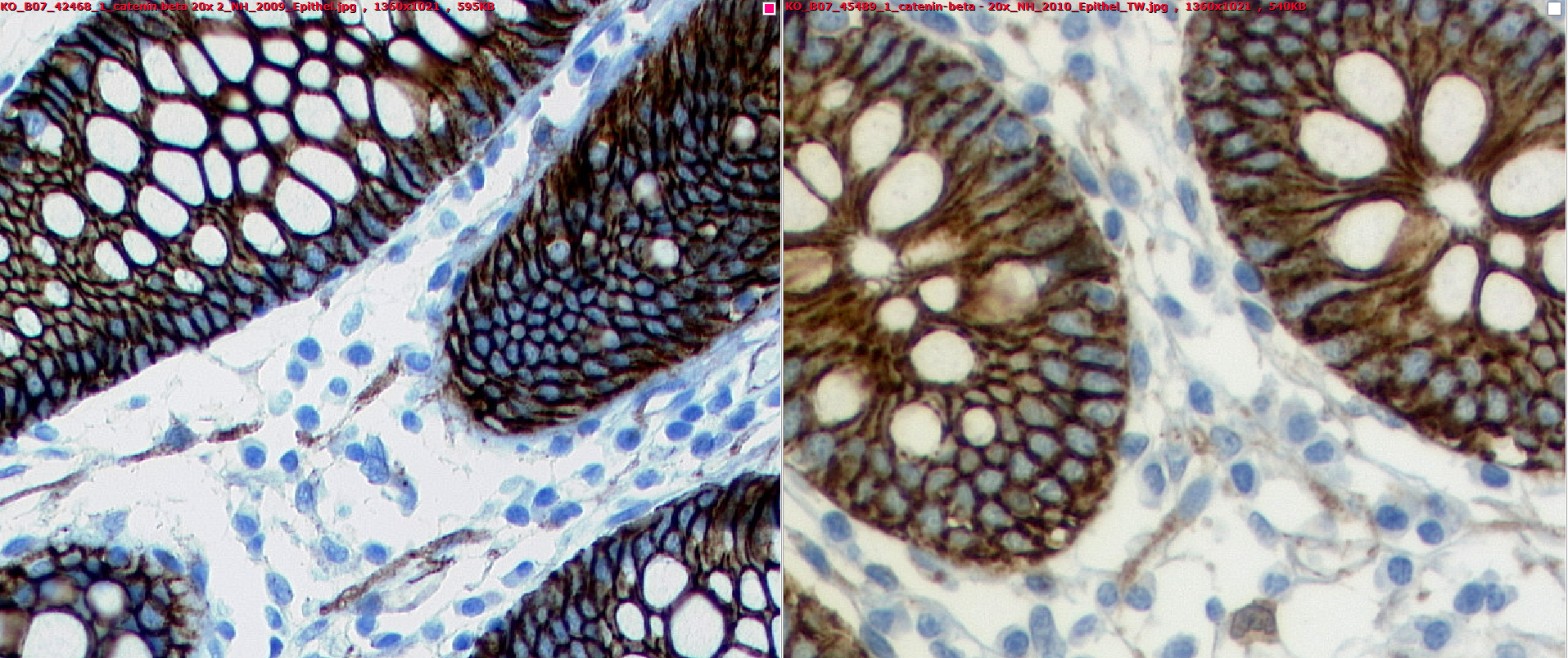


Statistical analysis

| **Number of cells** | **Number of**  **positive nuclei** | **Number of nuclei in %** | **Mean in %** |
| --- | --- | --- | --- |
| CD 1 : 60 | 37 | 61.67 | 52.78 |
| CD 2 : 60 | 30 | 50.00 |  |
| CD 3: 60 | 28 | 46.67 |  |
| Control 1 : 60 | 9 | 15.00 | 16.67 |
| Control 2 : 60 | 11 | 18.33 |  |
